# The quest for Homer’s *moly*: exploring the potential of an early ethnobotanical complex

**DOI:** 10.1186/s13002-024-00650-7

**Published:** 2024-01-20

**Authors:** Rafael Molina-Venegas, Rodrigo Verano

**Affiliations:** 1 Department of Ecology, Faculty of Science, Universidad Autónoma de Madrid, 28049 Madrid, Spain; 2 Biodiversity and Global Change Research Center (CIBC-UAM), Universidad Autónoma de Madrid, 28049 Madrid, Spain; 3 Department of Classical Philology, Universidad Complutense de Madrid, 28040 Madrid, Spain

**Keywords:** AChE-inhibiting properties, Amaryllidoideae, Ethnobotanical complex, Homer’s *moly*, Oral traditions, Phylogeny

## Abstract

The Homeric plant *moly* is a mysterious herb mentioned in Book 10 of the *Odyssey*. In the early 1980s, a pharmacological thesis to identify the plant was put forward for the first time, regarding the snowdrop (*Galanthus nivalis* L.) as candidate species. The proposal was inspired by the snowdrop’s acetylcholinesterase (AChE)-inhibiting properties and its alleged morphological reminiscence to other plants called *moly* by ancient Greek herbalists. Here, we draw from a compilation of literature from various disciplines, together with an understanding of the Homeric epic as a repository of information based on oral traditions, to (i) show that the assimilation of Homer’s *moly* to *Galanthus nivalis* is, at the very least, questionable and (ii) frame and support a new synthesis of the pharmacological thesis. We suggest that the uncertainty that revolves around the identity of Homer’s *moly* can be tied to an unnamed phylogenetic clade of closely related Mediterranean native species with AChE-inhibiting properties. Further, we speculate that Homer’s *moly* might represent an early record of an ethnobotanical complex, a sort of cultural taxon resulting from the cognitive crossbreeding of closely related taxonomic species that could have been interchangeably used due to their rough resemblance and common AChE-inhibiting properties. Such cultural taxon would have referred to the phytonym *moly* by the centuries-old oral traditions that ultimately crystallized in the poem. We also venture that sea daffodils (*Pancratium* spp.) could have greatly contributed to shaping the botanical archetype in the myth as we know it today.

**Supplementary Information:**

The online version contains supplementary material available at 10.1186/s13002-024-00650-7.

## Introduction

Homer’s *Odyssey* is one of the oldest epic poems of ancient Greek literature, still *au courant* among contemporary readers. The poem recounts the adventures of the Greek hero Odysseus during his journey back home from the Trojan War. Most scholars concur that the poem must have been first put into writing sometime in the eighth-century BC, in a form more or less similar to the one we read today, either by a single author—the blind bard Homer—or through several different textualization processes (see [1] for an overview). Regardless, the *Odyssey*—and the *Iliad*, the other major ancient Greek epic poem attributed to Homer—are clearly not the product of a single individuality but the outcome of a centuries-long oral tradition [2] (a collection of his papers published between 1928 and 1937) [3, 4]. It was the work of several generations of poets that gave birth to a corpus of myths and legends of times past, in which the exploits of gods and heroes were interwoven with all sorts of cultural and practical information to be transmitted orally from one generation to the next. Therefore, the *Iliad* and the *Odyssey* should not be read as mere literary pieces but also as repositories of social memory and knowledge built through patterns similar to those employed by other societies lacking the art of the written word [5]. From this perspective, and with the necessary caution, the poems can be approached as valuable sources of historical and ethnological information about the populations in which they arose and to whom they refer.

Thus, among the fanciful fictions of Odysseus’ encounters with eldritch creatures, brutal one-eyed giants, and powerful magicians, it is possible to find some day-to-day life experiences of the Greek folk of the pre-Archaic period. For example, much of the naval technology and shipbuilding methods described in the *Odyssey* can be applied to boats of the Late Bronze Age [6–8], and many of the aspects of sea life described in the poem seem faithfully based on real seafaring experience [9]. As a matter of fact, both the *Iliad* and the *Odyssey* are actual user’s guides on how to carry out all kinds of activities: there we learn how sacrifices to the gods are executed [10], how meetings between peer aristocrats are conducted, or how the army is organized for battle [11]—the way soldiers should dress, following a well-acknowledged bottom-to-top pattern, is repeated throughout the poems using virtually identical verses (*e.g.,* in the *Iliad*, Paris in Book 3, Agamemnon in Book 11, Patroclus in Book 16, Achilles in Book 19; cf. [12]— along with other indications referring to geographical, political, religious, and social issues. Thus, we must separate the possible historical realities from the fantastical elements of the myths to grasp the best possible understanding of ancient Greek culture as it is encoded in Homeric poems. One of those myths where there might be more than meets the eye refers to the Homeric plant *moly*, a mysterious herb with an alleged pharmacological activity that saved the lives of Odysseus and his crew from terrible doom.

In the Homeric story (Homer, *Odyssey* 10.35–406),^1^ Odysseus and his crew arrived at the mysterious island of Aeaea, home of the goddess-sorceress Circe. Once they landed, Odysseus climbed up to a rugged outlook point and beheld the island when he spotted a column of smoke rise through a thicket in the woods. Intrigued, Odysseus rejoined his crew and sent half of the men to find the source of the smoke. In doing so, they unwittingly ventured into the territory of Circe, the polypharmacist. The stone-made palace stood in the middle of a forest glade guarded by wolves and lions that, to the men’s amazement, did not rush upon them as the sorceress had bewitched the beasts using evil drugs. Circe came out of the palace bidding the men to enter, and they all accepted the invitation except Eurylochus, who suspected mischief and stayed outside. Circe’s lavish banquet soon became the scene of witchcraft, as she had mixed wicked drugs in the food to make the men forget their homeland entirely and had turned them into pigs—allowing them to keep their human reasoning faculties. Eurylochus hurried back to warn Odysseus, who urgently set out to rescue his men on his own. As he made his way through a grove toward Circe’s house, Odysseus encountered the god Argeiphontes (Hermes), who revealed the properties of the plant that would protect the Greek hero against Circe’s baneful mixture:

Homer, *Odyssey* 10.287–292

τῆ, τόδε φάρμακον ἐσθλὸν ἔχων ἐς δώματα Κίρκης.

ἔρχευ, ὅ κέν τοι κρατὸς ἀλάλκῃσιν κακὸν ἦμαρ.

πάντα δέ τοι ἐρέω ὀλοφώϊα δήνεα Κίρκης.

τεύξει τοι κυκεῶ, βαλέει δ' ἐν φάρμακα σίτῳ·

ἀλλ' οὐδ' ὧς θέλξαι σε δυνήσεται· οὐ γὰρ ἐάσει.

φάρμακον ἐσθλόν, ὅ τοι δώσω, ἐρέω δὲ ἕκαστα

Here, go to Circe’s house with this excellent drug,

which will protect your head from evil

Now I will tell you all of Circe’s deadly arts

She will make you a potion, mixing food with poison

But you will not be enchanted, because it will not let it

the excellent drug, which I will give you, and I will tell you all

After Argeiphontes warned Odysseus of the wiles that Circe was plotting, the narrative continues to describe the plant in Odysseus’ words:

Homer, Odyssey 10.302–306.

ὣς ἄρα φωνήσας πόρε φάρμακον ἀργειφόντης.

ἐκ γαίης ἐρύσας καί μοι φύσιν αὐτοῦ ἔδειξε.

ῥίζῃ μὲν μέλαν ἔσκε, γάλακτι δὲ εἴκελον ἄνθος·

μῶλυ δέ μιν καλέουσι θεοί, χαλεπὸν δέ τ' ὀρύσσειν.

ἀνδράσι γε θνητοῖσι· θεοὶ δέ τε πάντα δύνανται.

So saying Argeiphontes gave me the drug

drawing it from the earth, and showed me its nature.

It was black in the root, but the flower was like milk.

And the gods call it moly, and it is difficult to dig

for mortal men, but the gods can do anything.

Odysseus continued on his way to the house of Circe and encountered her. As foreseen by the god, the sorceress offered him a potion with a drug therein, but the mixture would have no effect this time. Odysseus drank it down, drew his sword, and rushed upon Circe, who, amazed that Odysseus was not bewitched, knelt before the Greek hero and cried in lamentation. Finally, Odysseus asked Circe to dispel the enchantment that changed his comrades into pigs, so they became men again.

The memory impairment and delusional and hallucinatory effects of some Mediterranean native plants, such as henbane (*Hyoscyamus* spp.) and mandrake (*Mandragora* spp.), appear to have been known by ancient Greeks. As such, most scholars agree that these plants were already used in Archaic Greece for their analgesic, anesthetic, and narcotic properties [14–16]. Indeed, it has been suggested that the Homeric *nepenthes*, the “drug of forgetfulness” mentioned in Book 4 of the *Odyssey* used by Helen to “bring forgetfulness” (Homer, *Odyssey*, 4.220–221 «αὐτίκ᾽ ἄρ᾽ εἰς οἶνον βάλε φάρμακον, ἔνθεν ἔπινον, / νηπενθές τ᾽ ἄχολόν τε, κακῶν ἐπίληθον ἁπάντων». *Inmediately she dropped some drug into the wine, of which they drunk; and the drug caused the disappearance of pain and discomfort, and the oblivion of afflictions*), may have contained henbane among other narcotic plants, including mandrake and opium poppy [16]. Moreover, the Renaissance physician Andrés de Laguna pointed out that the Greeks knew henbane as *hyoscyamo*—meaning “pig bean” [17]: 416–418—and Dioscorides, a Greek physician who lived in the first century AD, mentions mandrake (μανδράγορας) and points out that the plant is also referred to as *circea* (Κιρκαία) (Dioscorides, *De materia medica* 4.75.1.2), which may suggest a connection between these plants and the myth of Circe. Thus, their extremely toxic anticholinergic alkaloids [14] might have been fictitiously represented in the pharmacological composition of Circe’s “malignant mixtures” [15], which would explain Odysseus’ men’s forgetfulness and the hallucinatory state during which they believed they had been turned into pigs with human reasoning faculties. This pharmacological background inspired Plaitakis and Duvoisin [18] to propose that the plant that protected Odysseus from Circe’s witchcraft might not be magical but real, an actual acetylcholinesterase (AChE)-inhibiting antidote to reverse anticholinergic intoxication. Plaitakis and Duvoisin [18] argued that *Galanthus nivalis* L., a tiny European herb with AChE-inhibiting properties [19], fit the description of Homer’s *moly* and seemed reminiscent of botanical descriptions provided by ancient Greek herbalists in regard to other plants also called *moly* in Antiquity. Thus, Plaitakis and Duvoisin [18] contributed a convincing pharmacological argument for the first time to identify Homer’s *moly* as a real taxonomic species, and their proposal has remained widely accepted hitherto.

In this paper, we draw from a compilation of literature from various disciplines pertaining to systematic botany, phylogenetics, pharmacology, ethnobotany, biogeography, and ecology, together with an understanding of the Homeric epic as a repository of information based on oral traditions, to build upon the pharmacological thesis to identify Homer’s *moly* initiated by Plaitakis and Duvoisin [18] forty years ago. Firstly, we underline several issues concerning the identification of Homer’s *moly* as *Galanthus nivalis*. Then, we argue that, as long as the pharmacological thesis to identify Homer’s *moly* is accepted, the uncertainty that revolve around the plant can at least be tied to an unnamed phylogenetic clade of Mediterranean native species with AChE-inhibiting properties. Finally, we speculate that Homer’s *moly* might not represent a taxonomic species but an ethnobotanical complex of the time, that is, a set of different taxonomic species culturally recognized under the same phytonym due to shared properties [20], and we also venture that sea daffodils (*Pancratium* spp.) could have greatly contributed to inspire the tradition that ultimately crystallized in the version of the myth as we know it.

## The identification of Homer’s *moly* as *Galanthus nivalis*

In 1951, the Russian pharmacologist Mikhail Mashkovsky discovered that local villagers from the Urals used the wild snowdrop (*Galanthus* spp., most notably *Galanthus woronowii* Losinsk.) to stave off paralysis in children suffering from poliomyelitis [21]. The ethnobotanical use of the snowdrop in the Urals led to the isolation of galanthamine for the first time in 1952, an alkaloid with AChE-inhibiting properties [22]. Originally extracted from *Galanthus woronowii* and later from *Galanthus nivalis*, galanthamine was the subject of intense research in successive years for the treatment of neurological diseases such as Alzheimer [21] and Parkinson’s disease with dementia [23]. The use of *Galanthus nivalis* as a historical source of galanthamine, together with the promising AChE-inhibiting properties of the alkaloid [69], inspired the pharmacological arguments that Plaitakis and Duvoisin [18] put forward to connect *Galanthus nivalis* with Homer’s *moly*. The authors noted that *Galanthus nivalis* has white flowers and a bulb with dark brown outer scales [24], characteristics that fit well with the description of Homer’s *moly* in the *Odyssey*, that is, (white) “flower like milk” and a “black root” (Homer, *Odyssey*, 10.304). It is worth mentioning that early Greek botanists did not clearly distinguish between actual roots and bulbs, and they often referred to the latter as “round roots” (cf., for instance, Theophrastus, *Historia Plantarum* 6.6.8, 6.6.9, and 7.12.3 on the “roots” of κρίνον (*krinon*) [*Lilium* spp.], νάρκισσος (*narkissos*) [*Narcissus serotinus* L.], and φάσγανον (*phasganon*) [*Gladiolus italicus* Mill.], respectively^2^). On the other hand, color operated differently in Antiquity from what we are used to today [26, 27]. Thus, it should be no surprise that Homer’s *Odyssey* describes a bulb with dark brown outer scales as a “black root”. Fortunately, the poem provides a comparative element, describing the color of the flowers (i.e., “like milk”). Given that ancient Greeks obtained milk exclusively from goats and sheep—which produce certainly white milk, especially the former [28]—we can accept, without objection, that Homer’s *moly* bears white flowers.

Perhaps as a remote reflection of the medicinal practices carried out in ancient Greece, poisonous plants with anticholinergic properties are still used in many rural areas of the world as traditional remedies [76], where numerous accidental and deliberate anticholinergic intoxications have been reported [29, 30]. Thus, it is reasonable to think that the folks of ancient societies such as the Greeks of the early Dark Ages were also highly exposed to anticholinergic intoxication, hence the importance of having an AChE-inhibiting antidote at hand—allegedly *Galanthus nivalis* [18]. Indeed, the tradition of using poisonous plants as medicinal remedies in Greek Antiquity prevailed and was further developed by Roman and Byzantine scientists, who managed to mitigate their highly toxic effects in healing practices [31]. It is also worth remembering that the same word ‘phármakon’ (φάρμακον) is used indistinctly for poison, antidote, and medicine in ancient Greek.

The primary merit of Plaitakis and Duvoisin [18] was that their theory transcended morphological descriptions for the first time, thus setting the foundations for a new pharmacological perspective on the interpretation of the myth. The laudable but ill-advised historical attempts to identify Homer’s *moly* on the sole basis of morphological evidence—a total of 11 genera from 8 different families have been proposed as candidates (see [32] and references therein)—were finally left behind.

## Challenging the identification of Homer’s *moly* as *Galanthus nivalis*

Theophrastus, a Greek philosopher and scientist who lived between c. 371 – c. 287 BC, often considered the father of botanical science, provided the description of a plant called *moly* (Theophrastus, *Historia Plantarum* 9.15.7):

τὸ δὲ μῶλυ περὶ Φενεὸν καὶ ἐν τῇ Κυλλήνῃ. φασὶ δ' εἶναι καὶ ὅμοιον ᾧ ὁ Ὅμηρος εἴρηκε, τὴν μὲν ῥίζαν ἔχον στρογγύλην προσεμφερῆ κρομύῳ τὸ δὲ φύλλον ὅμοιον σκίλλῃ· χρῆσθαι δὲ αὐτῷ πρός τε τὰ ἀλεξιφάρμακα καὶ τὰς μαγείας· οὐ μὴν ὀρύττειν γ' εἶναι χαλεπόν, ὡς Ὅμηρός φησι.

- *The moly grows about the area of Pheneus and on Mt. Cyllene* [sc. the Eastern part of ancient Arcadia]. *It is said that this plant is also like* (ὅμοιον) *the moly that Homer mentioned, having a round root like an onion and a leaf like a squill* (ὅμοιον σκίλλῃ) *and that it is used against spells and magic arts. But not that it is difficult to dig up, as Homer says.*

**Fig. 1:**
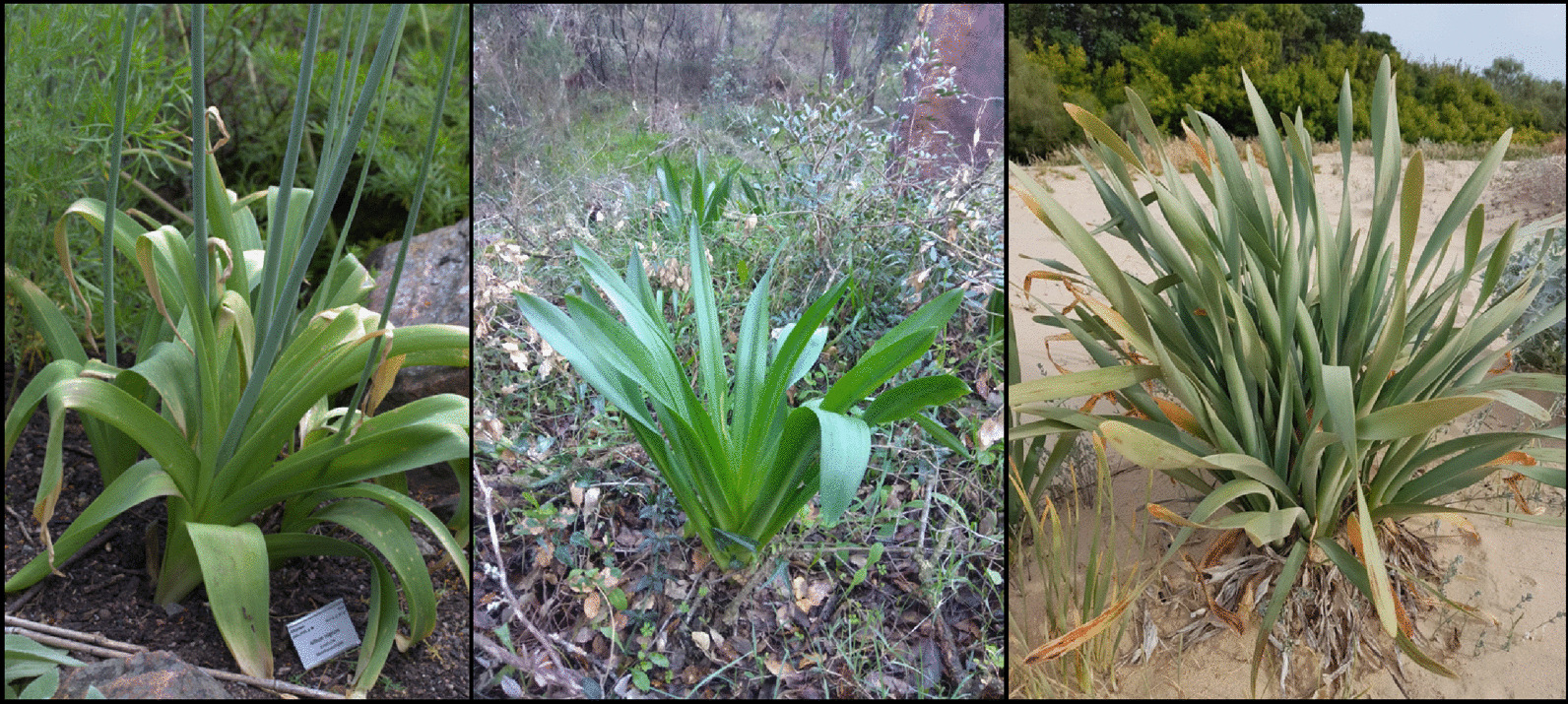
From left to right, leaves of broadleaf garlic (*Allium nigrum* L.), sea onion (*Drimia maritima* L. Stearn), and sea daffodil (*Pancratium maritimum* L.). Right, picture courtesy of Agustina Venegas Lagüens. Third party material: Figure: picture of *Allium nigrum* is protected under CC-BY 3.0 license (https://commons.wikimedia.org/wiki/File:Allium_nigrum_GotBot_2015_001.jpg) and is authored by Averater

Unquestionably, the *moly* referred to in the passage (hereafter “Theophrastus’ *moly*”) is not Homer’s *moly* but a different plant that, according to Theophrastus’ sources, resembles Homer’s *moly* in that it has “a round root like an onion” and “a leaf like a squill”. Plaitakis and Duvoisin [18] implicitly assumed that these traits are certainly shared between the plants, and they used this evidence to support their theory further. As such, they argued that the tiny leaves of species in the modern genus *Scilla* are similar to those of *Galanthus nivalis*. While we have no objection against the assumption that Theophrastus’ *moly* may resemble Homer’s *moly*, we note that the assimilation of the squill mentioned in the passage (“σκίλλῃ”) to a species in the genus *Scilla* is unjustified. Instead, Theophrastus seems to be referring to the sea onion—*Drimia maritima* (L.) Stearn*—*an interpretation that finds a certain consensus [33]. In his *De Materia Medica*, Dioscorides described ‘σκίλλα’ as a plant with a large onion (i.e., clearly distinguishing between the internal and external leaves) with multiple medicinal properties (Dioscorides, *De materia medica* 2.171.1–2). Later, Renaissance botanists did not hesitate to identify ‘σκίλλα’ as the sea onion [17]: 247, [34]: 289–292, also called ‘cebolla albarrana’ in Spanish. The Latin translation for ‘σκίλλα’ is *squilla*, hence the confusion in the misidentification of the squill mentioned in the passage as a species in the modern genus *Scilla*. Further, the leaves of the sea onion show a remarkable resemblance to those of broadleaf garlic (*Allium nigrum* L., Fig. 1), which led to the identification of Theophrastus’ *moly* as *Allium nigrum* and, more certainly, *Allium* sp. (see [32] and references therein). In sum, the passage about Theophrastus’s *moly* seems to confirm the bulbous nature of the Homeric plant, whose leaves would resemble those of the sea onion (*Drimia maritima*), according to Theophrastus’ sources.

Plaitakis and Duvoisin [18] also referred to Dioscorides’ account of a plant termed *moly* (cf. Dioscorides, *De materia medica* 3.47.1.1) to further support the identification of Homer’s *moly* as *Galanthus nivalis*. The passage in Dioscorides’ *De materia medica* reads as follows:

μῶλυ· τὰ μὲν φύλλα ἔχει ἀγρώστει ὅμοια, πλατύτερα δέ, ἐπὶ γῆν < κλώμενα > , ἄνθη < δὲ > λευκοΐοις παραπλήσια, γαλακτόχροα, ἥσσονα δὲ πρὸς τὰ τοῦ ἴου, καυλὸν δὲ λεπτόν, πήχεων τεσσάρων· ἐπ' ἄκρου δὲ ἔπεστιν ὡσεὶ σκορδοειδές τι· ῥίζα δὲ μικρά, βολβοειδής.

- Mol﻿y: *it has leaves similar to grass* (agrostis), *but flatter, lying on the ground, flowers similar to the white violet* (leukoion), *milky-colored, but smaller than those of the violet* (ion), *and the stem is thin, of four cubits; at the top part there is something as if it were garlic, and the root is small, bulb-like.*

Here, Plaitakis and Duvoisin [18] assumed that *leukoion* (λευκόϊον) in Dioscorides’ passage refers to the modern genus *Leucojum*, whose species show a great resemblance to *Galanthus nivalis* but with larger flowers [35]. The authors used such flower-size comparison as evidence that the description of Dioscorides’ *moly* is consistent with the appearance of *Galanthus nivalis.* However, Dioscorides describes the plant *leukoion* as follows (Dioscorides *De materia medica*, 3.123.1.1):

λευκόϊον γνώριμον. ἔστι δ’ αὐτοῦ διαφορὰ ἐν τῷ ἄνθει· ἢ γὰρ λευκὸν ἢ μήλινον ἢ πορφυροῦν εὑρίσκεται.

Leukoion: *a very well-known plant; its flowers are very different, as they are white, yellow, and purple.*

Different authors have interpreted Dioscorides’ *leukoion* as a phytonym encompassing two morphologically similar species in the Brassicaceae family: the gillyflower (*Matthiola incana* (L.) W.T.Aiton), which displays purple, violet, or white flowers [36], and the wallflower (*Erysimum cheiri* (L.) Crantz), characterized by its yellow or orange flowers, often tinged with purple [37]. This collective identification, involving species with flower color polymorphism, is consistent with Dioscorides’ definition of *leukoion* [35, 38, 39] and challenges the assumption that the *leukoion* in the passage is attributable to the modern genus *Leucojum*, as the latter exclusively comprises species with white flowers. As for the use of the phytonym in other Greek sources, it is worth noting that although Theophrastus also used the term *leukoion* in several passages (cf. HP 4.7.8, 6.8.1, 7.8.3), he did not make any reference to the color of the flowers in doing so. Therefore, attributing the term *leukoion* to describe snowflakes (*Leucojum* spp.) directly to Theophrastus or Dioscorides appears unjustified. It was not until several centuries later that Linnaeus adopted a Latinization of this term to designate these species [35]. Dioscorides’ *moly* has been usually regarded as *Allium sp*. (possibly *Allium nigrum*) with no objection to this generic identification (see [32], and references therein). However, Plaitakis and Duvoisin [18] argued that this interpretation is clearly erroneous because the flowers of *Allium* show no resemblance to those of *Leucojum*. It must be noted that Plaitakis and Duvoisin’s objection [18] is due to their inaccurate interpretation of Dioscorides’ *leukoion* as an actual species of *Leucojum*, and therefore it should be rejected. It follows that Dioscorides’ *moly* is irrelevant for the identification of Homer’s *moly*.

Another argument that Plaitakis and Duvoisin [18] used to support the identity of Homer’s *moly* as *Galanthus nivalis* concerns the species’ preferred habitat. According to the authors, the god *Argeiphontes* picked the plant *moly* out of the “moist sheltered ground” [18] that characterizes a “forest glen” (their proposed translation of “βήσσα” in Homer, *Odyssey* 10.275, following R. Lattimore’s translation of the same term a few lines before, in 10.210, as “glens”), a habitat description that fits well to the ecological preferences of *Galanthus nivalis* [24]. However, this translation should be taken with caution since the etymology of ‘βήσσα’ is still unresolved—no clear cognates have been proposed so far [40]: 212–213)—and the word has little use outside the Homeric corpus. English translators render the form using a variety of terms—cf. the passage in question: ‘glades’ [41], ‘grove’ [42], ‘glens’ [43], again ‘glades’ [44]—pointing to a certain consensus that it refers to a wooded area. Indeed, in the passage, the name appears described with the adjective ‘sacred’ (ἱεράς), which is fitting for a forest or grove. Furthermore, the frequent use of the word in the formula “οὔρεος ἐν βήσσῃς” (usually translated as “in the mountain glens”) suggests a connection with mountain topography (οὔρεος being the genitive case in epic language of the noun ὄρος, the Greek word for ‘mountain’). However, reducing the referent of βῆσσα in the passage to a “moist sheltered ground” (as [18] do, emphasizing the moist quality of the habitat) seems unjustified in light of the evidence from the Greek text and language. Besides, moist forest habitats are relatively rare in the Mediterranean, where drought-tolerant tree species and tall shrubs dominate the woodlands that characterize the region [45]. Indeed, *Galanthus nivalis* is a species of Euro-Siberian affinity [46] with only a few scattered populations in humid refugia across the Mediterranean [47]. This inconspicuous distribution may have inspired Plaitakis and Duvoisin [18] to propose that the antidote would be difficult to find in the Mediterranean. However, modern pharmacological accounts have revealed the existence of few Mediterranean species with similar or even stronger AChE-inhibiting properties than *Galanthus nivalis* (Table S1), which also fit well with the description of Homer’s *moly*.

## Homer’s *moly* as an ethnobotanical complex?

Homer’s epics mention a relatively large number of plants [48] with acknowledged medicinal uses in Minoan, Mycenaean, and Egyptian Assyrian pharmacotherapies [49], which evinces pre-Archaic Greek society’s interest in the surrounding flora and its properties. This interest is hardly surprising since the drive for environmental knowledge is a cultural universal, and oral traditions have been shown to be very productive in transmitting large masses of in-depth practical knowledge about animals and plants [5]. It is, however, reasonable to think that, even though orally transmitted classifications can reach a high degree of complexity, the tradition of recognizing species as individual entities with specific characteristics was not established until the foundation of philosophical botany by Aristotle and, especially Theophrastus, in the fourth-century BC when the written culture was already widespread in ancient Greece. Previously, the classification of known species would be largely conditioned by a utilitarian perspective [50]. This botanical knowledge was built on empirically based plant lore passed on by word-of-mouth as ‘memes’ of information—units for carrying cultural ideas, symbols, or practices [51]—that ultimately crystallized into successful oral traditions such as the ones that converged in Homer’s *Odyssey*. In the particular case of our *moly*, it is not difficult to imagine that the widespread use of the effective but risky home-made anesthetics and sedatives of the time (henbane and mandrake, allegorically represented as “Circe’s malignant drugs”) could eventually lead to the unpleasant consequences of unintended anticholinergic intoxication (forgetfulness and impeding hallucinatory states represented as “men turned into pigs”). Hence, the importance of having an AChE-inhibiting antidote at hand (the plant *moly* granted to Odysseus). Given that the times when the poem is contextualized date back to well before the emergence of philosophical botany and its taxonomic identification methodologies, we propose that ancient Greeks of the time may have used several species with AChE-inhibiting activity interchangeably due to their similar pharmacological activity and general appearance (white flowers and bulbs with dark-colored outer scales).

**Fig. 2:**
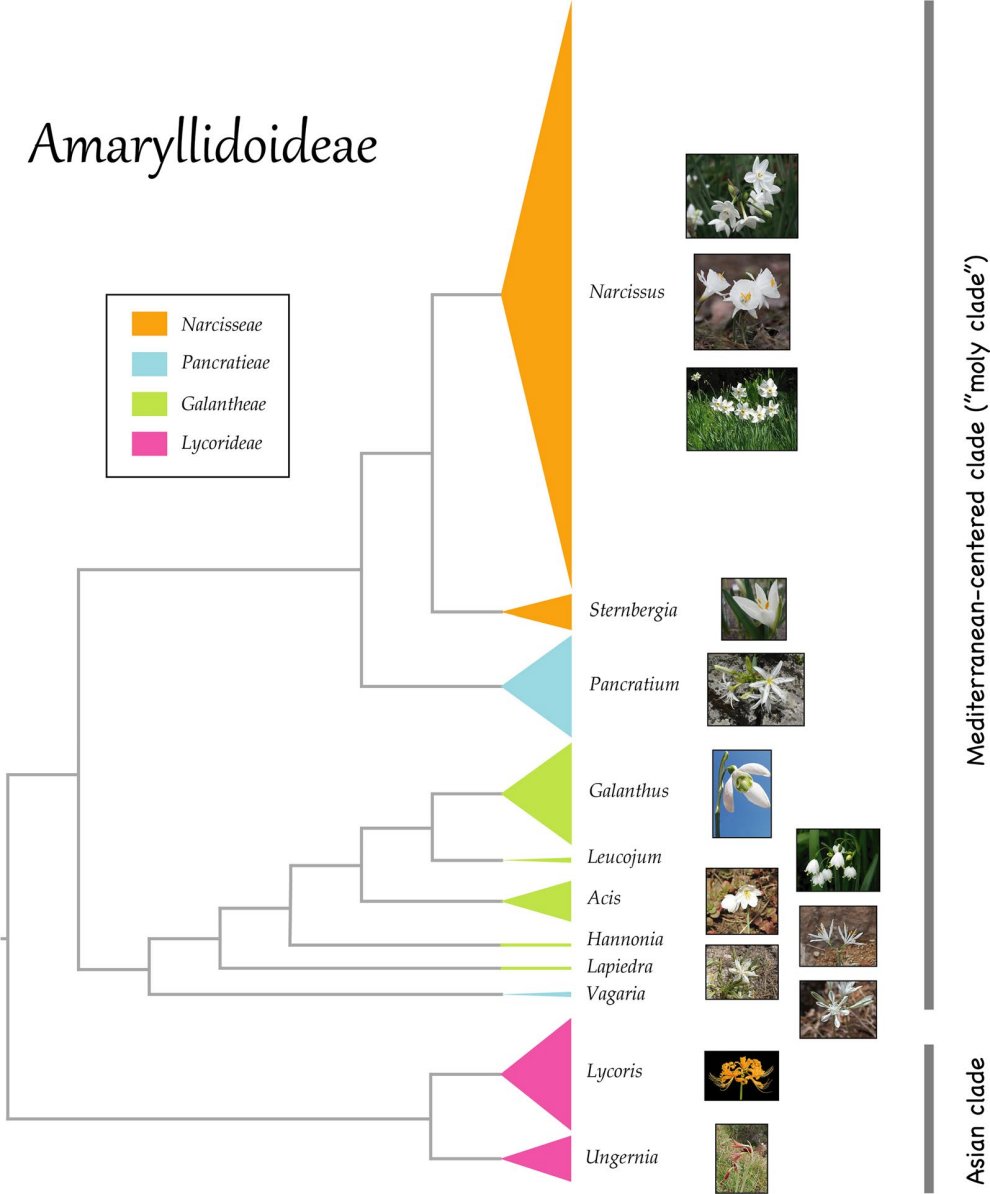
Genus-level phylogeny of the subfamily Amaryllidoideae obtained from Smith and Brown [73]. The lineage splits between the tribe Lycorideae in Central and East Asia (bottom, “Asian clade”) and a Mediterranean-centered unnamed clade (top, here “*moly* clade”) composed of the tribes Galantheae, Narcisseae, and Pancratieae. The *moly* clade includes many species with white flowers, bulbs with dark-colored outer scales, and reported AChE-inhibiting activity (Table S1). The size of the triangles is proportional to the number of species in each genus according to WFO [74]. Pictures from top to bottom: *Narcissus papyraceus* Ker Gawl., *Narcissus cantabricus* DC., *Narcissus poeticus* L., *Sternbergia candida* B.Mathew & T.Baytop, *Pancratium Illyricum* L., *Galanthus nivalis* L., *Leucojum aestivum* L., *Acis valentina* (Pau) Lledó, A.P.Davis & M.B.Crespo, *Hannonia hesperidum* Braun-Blanq. & Maire, *Lapiedra martinezii* Lag., *Vagaria parviflora* Herb., *Lycoris aurea* (L’Hér.) Herb., and *Ungernia sewerzowii* (Regel) B.Fedtsch. Figure: Picture of *Narcissus papyraceus* is protected under CC-BY 2.0 license (https://commons.wikimedia.org/wiki/File:Narcissus_white.jpg) and is authored by Juni; picture of *Narcissus cantabricus* is protected under CC-BY-NC-SA 2.0 license (https://www.flickr.com/photos/84259756@N05/15982337613) and is authored by Mark Gurney; picture of *Narcissus poeticus* is protected under CC-BY-SA 3.0 license (https://commons.wikimedia.org/wiki/File:Narcissus_poeticus_%27Recurvus%27.jpg) and is authored by Meneerke bloem; picture of *Sternbergia candida* is protected under CC-BY-SA 3.0 license (https://commons.wikimedia.org/wiki/File:Sternbergia_candida1a.UME.jpg) and is authored by Epibase; picture of *Galanthus nivalis* is protected under CC-BY-SA 2.0 license (https://commons.wikimedia.org/wiki/File:Galanthus_nivalis_2005.jpg) and is authored by Darkone; picture of *Leucojum aestivum* is protected under CC-BY-SA 3.0 license (https://commons.wikimedia.org/wiki/File:Leucojum_aestivum_2010_4×3.jpg) and is authored by Hans Bernhard; picture of *Acis valentina* is protected under CC-BY-SA 4.0 license (https://commons.wikimedia.org/wiki/File:Acis_valentina_flower.jpg) and is authored by Meneerke bloem; picture of *Hannonia hesperidum* is protected under CC-BY-NC 4.0 license (https://www.teline.fr/en/photos/amaryllidaceae/hannonia-hesperidum) and is authored by Xavier Bassières; picture of *Lapiedra martinezii* is protected under CC-BY-SA 4.0 license (https://commons.wikimedia.org/wiki/File:Lapiedra_martinezii_-_Flor_de_la_estrella.jpg) and is authored by Formater; picture of *Vagaria parviflora* is protected under CC-BY 3.0 license (https://commons.wikimedia.org/wiki/File:Pancratium_parviflorum_flower_1.JPG) and is authored by Gideon Pisanty; picture of *Ungernia sewerzowii* is protected under CC-BY-SA 4.0 license (https://commons.wikimedia.org/wiki/File:Ungernia_sewerzowii_31659963.jpg) and is authored by Kudaibergen Amirekul

Natural AChE-inhibiting compounds are widely distributed across the plant phylogeny, including representative species from at least 40 families [52, 53]. Of this pool, Amaryllidoideae (Amaryllidaceae s.s.) is the leading group, contributing about a dozen genera with species for which strong AChE-inhibiting activity has been reported worldwide [53, 54]. The Eurasian lineage of Amaryllidoideae splits between the tribe Lycorideae in Central and East Asia and its sister clade, a Mediterranean-centered (including the territories around the shore of the Black Sea) unnamed clade composed of the tribes Galantheae, Narcisseae, and Pancratieae [55] (Fig. 2). Remarkably, AChE-inhibiting activity has been reported in seven out of nine genera in the latter clade (Table S1), all of which include species with white flowers and bulbs with dark-colored outer scales. This evolutionary pattern supports the idea that Homer’s *moly* may encompass a group of closely related species (hereafter, “*moly* clade”) that ancient Greeks could have used indistinctly as sources of an AChE-inhibiting antidote. Numerous examples of taxonomic (and often phylogenetically related) species that are recognized under the same cultural phytonym for their shared morphological and medicinal features lend credibility to this hypothesis [20]. For example, the phytonym ‘Mirto’ refers to five red-flowered, perennial, aromatic, and culturally important *Salvia* (subgenus *Calosphace*) species that are used indistinctly to treat the folk-illness *susto* and other disorders in Mexico [56], ‘Árnica’ is a complex of popular medicinal species in the Iberian Peninsula and Balearic Islands (24 out of 32 in the Asteraceae family), most of which are yellow-flowered herbaceous perennial plants with shared therapeutic uses [57], and the Spanish phytonym ‘manzanilla’ or ‘camomila’ refers to more than sixty similar species in the Asteraceae, including some of the most popular ingredients of digestive beverages in Spain along with their substitutes and adulterants [58]. Thus, we hypothesize that Homer’s *moly* may represent an early record of an ethnobotanical complex, a group of closely related species with similar AChE-inhibiting properties and morphological traits primarily distributed throughout the Mediterranean Basin. The interchangeable medicinal use of the species and their distinctive appearance (bulbous species with dark-colored scales and white flowers are not particularly numerous in the Mediterranean) would justify the vague description of Homer’s *moly* that has come down to us.

## Venturing an archetypal label taxon for Homer’s *moly* ethnobotanical complex

**Fig. 3:**
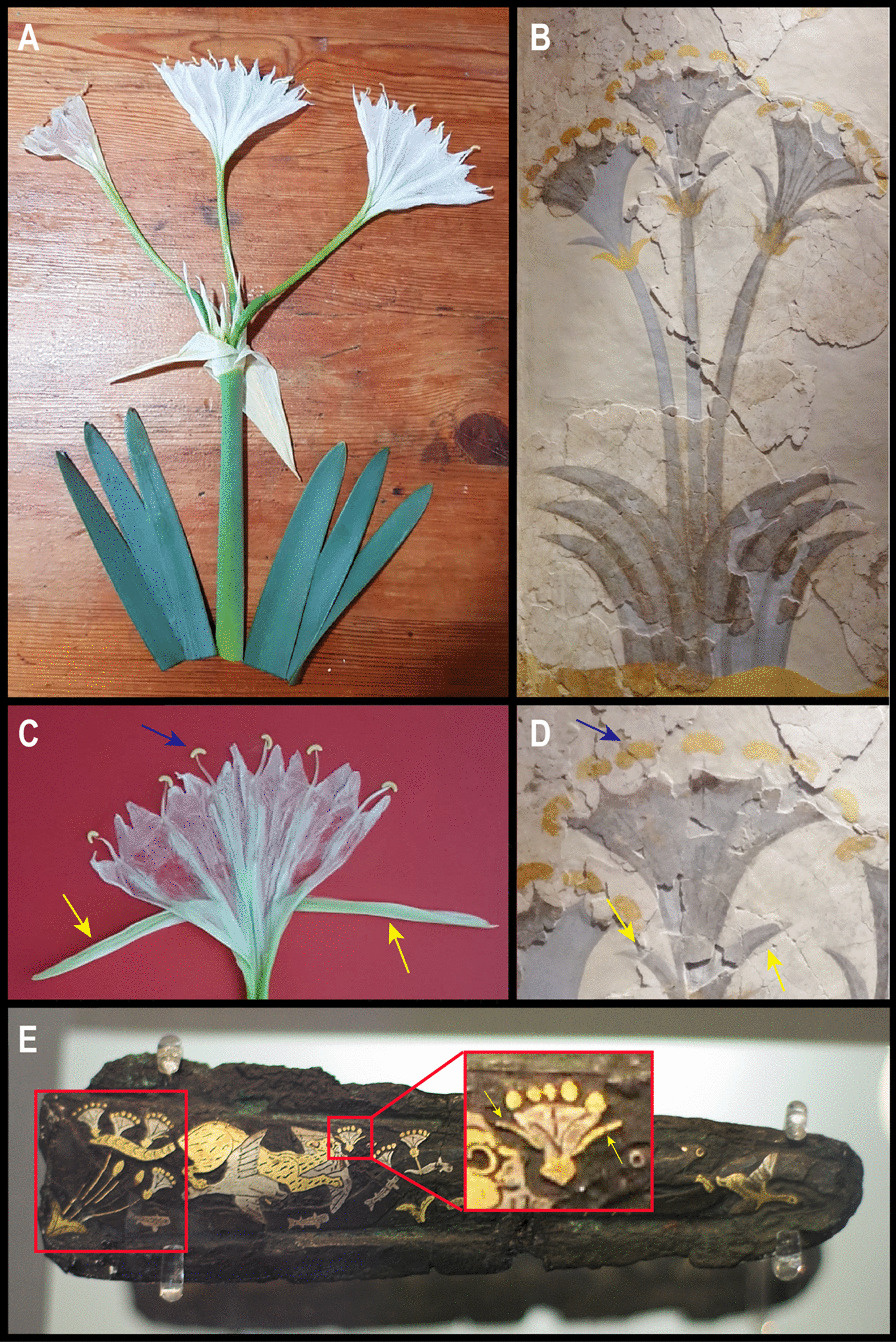
Sea daffodil, *Pancratium maritimum* L., depicted in a fresco section of the House of the Ladies in Akrotiri (**b**, **d**) and a Mycenaean bronze blade (**e**). The top-left picture (**a**) shows a *Pancratium maritimum* specimen that was mounted to look like the plant in the fresco by opening the bell-shaped corona on one side and removing the outer parts of the perianth (tepal lobes). The middle-left image (**c**) shows the details of a sea daffodil flower with six tepal lobes of which two were left attached (yellow arrows) to increase the resemblance with the flowers depicted in the fresco (**d**) and the bronze blade (**e**). The similarity in the banana-like anthers between the real specimen and the fresco (blue arrows), where they are depicted as seven instead of six (possibly due to the magic associated with this number across many traditions), is astonishing. Note that unrolling, omitting, and modifying floral elements for the purpose of stylization in ornamental reproduction is a typical feature of the two-dimensional ancient Greek painting [75]. Pictures (**a**) and (**c**) courtesy of Agustina Venegas Lagüens. Figure: picture (**b**) is protected under CC-BY 3.0 license (https://commons.wikimedia.org/wiki/File:Prähistorisches_Museum_Thira_Papyrusfresko_03.jpg) and is authored by Olaf Tausch; picture (**e**) is protected under CC-BY-SA 3.0 license (Bronze Blade: https://commons.wikimedia.org/wiki/File:Dagger_inlaid_Mycenaean_16_c_BC,_NAMA_765_102881.jpg) and is authored by Zde

Ethnobotanical complexes can sometimes be labeled by a single dominant species that shows the highest availability, publicity, and/or effectiveness relative to more local plants [20, 59], and the species in the *moly* clade greatly differ in their distribution and abundance within the Mediterranean basin. For example, while some species with documented AChE-inhibiting activity are rare (e.g., *Galanthus nivalis*), sea daffodils (*Pancratium spp.*) are abundant and widespread in the Mediterranean. Indeed, archeological records show that sea daffodils were already known in the pre-Homeric Aegean area. As such, they are spectacularly depicted on the Minoan frescos of the House of the Ladies in Akrotiri (c. seventeenth-century BC) and appear sculpted in a Mycenaean bronze blade [60, 61] (Fig. 3). Although these archeological records do not provide direct evidence of the medicinal use of sea daffodils among ancient Greeks, it is, however, likely that their recurrent contact with the plants was accompanied by the examination of their properties, a piece of knowledge that could have ended up in the story of Homer’s *moly* as a result of oral traditions. Notably, sea daffodils grow on beaches and coastal sand dunes across the entire Mediterranean basin and some parts of the Black Sea [62]; hence, it is reasonable to think that ancient Greeks, as seafaring people, may have encountered these plants regularly.

**Fig. 4:**
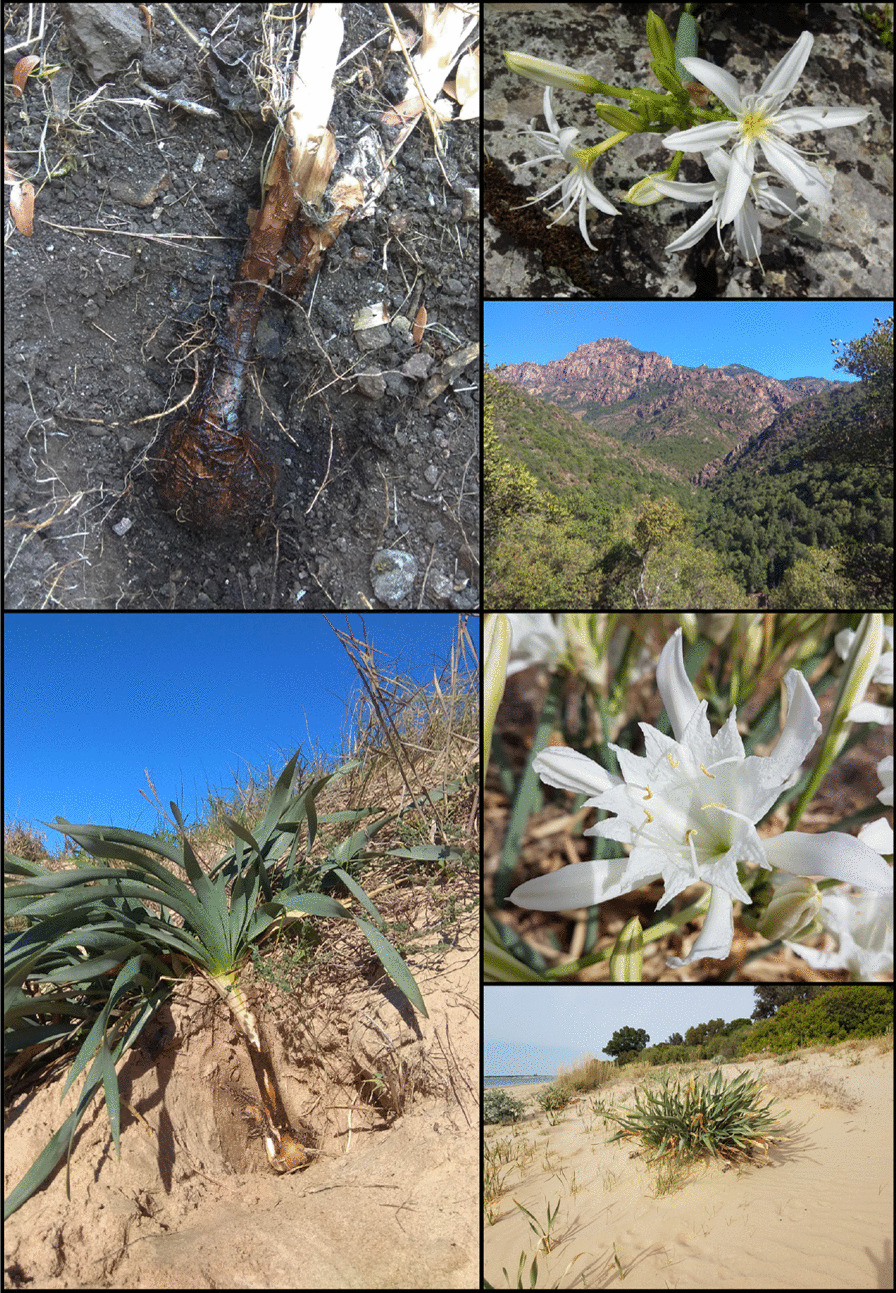
Top images: bulb, flowers, and typical woodland habitat (Gorges de la Specunca, Corsica) of the Illyrian sea daffodil (*Pancratium Illyricum* L.). Bottom images: bulb, detail of the flower, and typical coastal sand dune habitat (Sanlúcar de Barrameda, Spain) of the sea daffodil (*Pancratium maritimum* L.). Note the deeply buried bulbs with dark outer scales and the white flowers of both species. Top left picture courtesy of Miriam Ugidos del Barrio and bottom right pictures courtesy of Agustina Venegas Lagüens

A detailed analysis of the passage of Theophrastus’ *moly* (Theophrastus, *Historia Plantarum* 9.15.7) may establish another link between sea daffodils and Homer’s *moly*. According to Theophrastus’ sources, the plant under consideration in the passage resembles Homer’s *moly* in that it has “a leaf like a squill” (τὸ δὲ φύλλον ὅμοιον σκίλλῃ). This description logically connects the shape of the leaves of the three plants mentioned, broadleaf garlic (*Allium nigrum*, Theophrastus’s *moly*), sea onion –*Drimia maritima*, the squill of the Greeks (see section "Challenging the identification of Homer’s moly as Galanthus nivalis"), and Homer’s *moly*. We interestedly noted that the shape of the leaves of the two identified species shows a great resemblance to that of sea daffodils (Fig. 1). Further, the passage reads that the plant under consideration “is not”, as Homer says, “difficult to dig up” (οὐ μὴν ὀρύττειν γ’ εἶναι χαλεπόν, ὡς Ὅμηρός φησι). This final remark is critical because it suggests that there is something in Homer’s *moly* that makes the plant difficult to dig up besides its bulbous nature. The contractile roots of sea daffodils can drag the bulbs down to depths up to 160 cm [63], which can certainly make the plants very difficult to unearth (Fig. 4). This distinctive feature of sea daffodils did not go unnoticed by late Enlightenment naturalists, who already described the plants as having “a large bulb, covered with a dark skin, sending out many thick strong fibers, striking deep in the ground” [64]. However, previous attempts to identify Homer’s *moly* paid little attention to this distinctive trait, perhaps because it was understood as fictitious by early *moly* hunters.

Against our hypothesis, it can be argued that if the place where Argeiphontes encountered Odysseus is to be considered in literal terms, sea daffodils may not be the best fit because they typically grow in coastal sand dunes that are hardly found inland [62]. In contrast, the poem recalls that Odysseus had moved inland through the glade, when the encounter with Argeiphontes took place (Homer, *Odyssey* 10.275). Although most modern scholars argue that any attempt at realistic geographic identification of Odysseus’ landfalls is in vain as they would represent imaginary places, our approach to Homer’s *moly* relies on the idea that at least certain tokens of factual information concerning the plant must somehow underlie the mythical narrative, and the description of its habitat seems relevant enough. Therefore, the ecological criticism of sea daffodils as candidates for Homer’s *moly* is legitimate. Interestingly, while most Mediterranean species in the genus are restricted to either sunny coastal areas (*Pancratium maritimum* L., *Pancratium foetidum* Pomel, *Pancratium arabicum* Sickenb.) or deserts (*Pancratium sickenbergeri* Asch. & Schweinf. ex Boiss.) [62], we noted that the Illyrian sea daffodil (*Pancratium illyricum* L.) diverges ecologically from its Mediterranean congenerics. The Illyrian sea daffodil is a Tyrrhenian endemism native to the islands of Corsica, Sardinia, Capraia, and Elba—territories already known to the Mycenaeans, as proven by archeological evidence [65]—where it grows from sea level to the montane zone in sunny or shady environments, including woodland areas [66]. It produces a potent acetylcholinesterase inhibitor [67], and, contrary to its Mediterranean congenerics, the ecological requirements of the Illyrian sea daffodil fit well with the description of the site where Odysseus was granted with the plant *moly* (if we assume that Circe’s Island is a geographically recognizable Mediterranean island dominated by woodlands, such as Corsica). Finally, the fact that some ancient Greek sources pinpointed Circe’s home to this area supports this hypothesis. Theophrastus (*Historia Plantarum* 9.15.1) mentions that the goddess dwells in the Tyrrhenian islands, and the Alexandrian poet Apollonius of Rhodes places her home somewhere south of the island of Elba (one of the islands where the Illyrian sea daffodil is endemic) in his *Argonautica* (4.660), an epic poem that drew from multiple ancient sources, including the *Odyssey*.

Be that as it may, the idea that sea daffodils were a major source of antidote for anticholinergic intoxication among Archaic Greeks seems reasonable and circumvents the problem of the geographic inconsistency of the *Odyssey* regarding the location of Circe’s Island because these plants are abundant and widespread throughout the entire Mediterranean basin and the shore of the Black Sea (particularly *Pancratium maritimum*). However, we must not lose sight of the fact that the myth of Homer’s *moly* likely resulted from the interaction between multiple orally transferred traditions that progressively converged toward the canonic version of the story as we know it today [3]. We must assume that many of these traditions have been lost forever, while others may have been consciously or unconsciously modified according to local circumstances as the story was repeatedly performed [68]. In this vein, we propose that Homer’s *moly* can be approached as a sort of cultural taxon resulting from the cognitive crossbreeding of multiple oral traditions, all of which are built based on closely related species that roughly resemble each other in their external appearance and AChE-inhibiting properties.

## Conclusions

The Greek myths likely encapsulate remnants of ancient knowledge, and objections to the pharmacological thesis of Homer’s *moly*, as proposed by Plaitakis and Duvoisin [18], are scarce aside from its speculative nature. However, we contend that associating the Homeric plant with *Galanthus nivalis*, a rare herb in the Mediterranean, is, at the very least, questionable. Recent accounts have unveiled a few phylogenetically related Mediterranean species that fit well with the description of Homer’s *moly* and share similar or even stronger AChE-inhibiting properties than *Galanthus nivalis*. Thus, accumulating evidence prompts a reconsideration of the initial proposal.

We speculate that, if we accept the pharmacological thesis of Homer’s *moly*, the plant might not signify a taxonomic species recognized in modern botany. Instead, it could represent an early record of an ethnobotanical complex—a cultural taxon resulting from the cognitive amalgamation of phylogenetically related Mediterranean-centered species. These species could have been interchangeably used due to their rough resemblance and common AChE-inhibiting properties. Early *moly* hunters seemingly overlooked the distinctive trait that makes the Homeric plant challenging to unearth, apart from its bulbous nature. This detail, coupled with indirect archeological evidence, suggests that sea daffodils (*Pancratium *spp.) could have significantly influenced the botanical archetype in the canonical version of the myth.

While we acknowledge that the theory of Homer’s *moly* as an ethnobotanical complex is as speculative as the pharmacological thesis it builds upon, numerous instances of taxonomic species recognized under the same cultural phytonym for their shared morphological and medicinal uses lend credibility to our hypothesis. The odyssey from the Homeric era to the present day continues with the introduction of a new perspective of Homer’s *moly* as an early ethnobotanical complex, a tradition likely transmitted orally among ancient Greeks that eventually solidified into the myth of Odysseus, Circe, and the plant *moly* as we know it today.

### Supplementary Information

**Additional file 1. Table S1.** Native species of Mediterranean Amaryllidoideae (including the territories around the shore of the Black Sea) with white flowers, bulbs with dark-colored outer scales and reported AChE-inhibiting activity.

## Data Availability

Not applicable.

## Abbreviation

AChE: Acetylcholinesterase

## Footnotes

1 Passages from Homer and other ancient literary sources are quoted throughout this paper according to the conventions of classical scholarship, that is, using author, title, book, and verse number (or book, chapter, and line number in prose authors). The text of the *Odyssey* is from the edition of [13], other texts of ancient cited authors (*sc*. Theophrastus [70], Apollonius [71], Dioscorides [72])) are likewise listed in the reference list under their editors’ names. The English translations of the *Odyssey,* as well as other ancient technical or literary works, have been produced directly from Greek by the authors of this paper and are intended to be as literal as possible.

2 The identification of the Greek phytonisms κρίνον (*krinon*), νάρκισσος (*narkissos*), and φάσγανον (*phasganon*) is based on William Thiselton-Dyer’s criterium in Arthur Hort’s translation of *Historia Plantarum*, *Enquiry into Plants* [70], which has been maintained in later translations of the work (e.g., [25].

## References

[1] Ready JL (2019). Orality, textuality, and the homeric epics. An interdisciplinary study of oral texts, dictated texts, and wild texts.

[2] Parry M (1971). The making of Homeric verse: the collected papers of Milman Parry.

[3] Foley JM, Morris I, Powell BB (2011). Oral tradition and its implications. A new companion to homer.

[4] Lord AB (1960). The singer of tales.

[5] Kelly L (2015). Knowledge and power in prehistoric societies.

[6] Casson L (1964). Odysseus’ Boat (Od. vol 244–257). Am J Philolos.

[7] Georgiou HS. Bronze Age sailing and Homeric evidence. In: Korres GS, Karadimas N, Flouda G, editors. Archaeology and Heinrich Schliemann: a century after his death: assessments and prospects. Athens; 2012. p. 523–9.

[8] Mark S (1991). Odyssey 5.234–53 and Homeric ship construction: a reappraisal. Am J Archaeol.

[9] Mann R (2019). Seafaring practice and narratives in Homer's Odyssey. Antichthon.

[10] Hitch S (2009). King of sacrifice: ritual and royal authority in the Iliad (Hellenic Studies Series 25).

[11] van Wees H, Morris I, Powell BB (2011). Homeric Warfare. A new companion to Homer.

[12] Reitz C. Arming scenes, war preparation, and spoils in ancient epic*.* In: Reitz C, Finkmann S, editors. Structures of epic poetry: Vol. I: Foundations. Vol. II.1/II.2: Configuration. Vol. III: Continuity. Berlin/Boston, De Gruyter; 2019. p. 13–38. 10.1515/9783110492590-024

[13] Allen TW. Homeri Opera (vol. III, Odyssey I-XII, 1917; vol. IV, Odyssey XIII–XXIV, 1919). Oxford University Press, Oxford; 1917–1919.

[14] Alizadeh A, Moshiri M, Alizadeh J, Balali-Mood M (2014). Black henbane and its toxicity: a descriptive review. Avicenna J Phytomed.

[15] Carod-Artal FJ (2013). Psychoactive plants in ancient Greece. Neurosci Hist.

[16] Hocking GM (1947). Henbane healing herb of Hercules and of Apollo. Econ Bot..

[17] Laguna A. Pedacio Dioscórides Anazarbeo. Acerca de la materia medicinal, y de los venenos mortíferos. Salamanca; 1563.

[18] Plaitakis A, Duvoisin RC (1983). Homer’s moly identified as *Galanthus Nivalis* L.: physiologic antidote to stramonium poisoning. Clin Neuropharmacol.

[19] Berkov S, Cuadrado M, Osorio E, Viladomat F, Codina C, Bastida J (2009). Three new alkaloids from *Galanthus nivalis* and *Galanthus elwesii*. Planta Med.

[20] Linares E, Bye R (1987). A study of four medicinal plant complexes of Mexico and adjacent United States. J Ethnopharmacol.

[21] Heinrich M, Lee Teoh H (2004). Galanthamine from snowdrop—the development of a modern drug against Alzheimer’s disease from local Caucasian knowledge. J Ethnopharmacol.

[22] Heinrich M, Cordell GA (2010). Galanthamine from *Galanthus* and other Amaryllidaceae–Chemistry and biology based on traditional use. The alkaloids: chemistry and biology.

[23] Aarsland D, Hutchinson M, Larsen JP (2003). Cognitive, psychiatric and motor response to galantamine in Parkinson’s disease with dementia. Int J Geriatr Psychiatry.

[24] Aedo C, Aedo C, Herrero A, Quintanar A (2013). *Galanthus* L. Flora iberica, 20.

[25] Díaz-Regañón JM. Theophrastus. Historia de las plantas. Biblioteca Clásica Gredos, Madrid; 1988.

[26] Bradley M, Butler S, Purves A (2013). Colour as synaesthetic experience in antiquity. Synaesthesia and the ancient senses.

[27] Sassi MM, Destrée P, Murray P (2015). Perceiving colors. A companion to ancient aesthetics.

[28] Sheehan JJ, Patel AD, Drake MA, McSweeney PLH (2009). Effect of partial or total substitution of bovine for caprine milk on the compositional, volatile, non-volatile and sensory characteristics of semi-hard cheeses. Int Dairy J.

[29] Serrano R (2018). Toxic plants: knowledge, medicinal uses and potential human health risks. Environ Ecol Res.

[30] Street R, Cele N, Mhlongo S, Baijnath H (2019). Poisonous plant exposure, human health harms and the development of culturally relevant public education messaging. Environ Epidemiol.

[31] Ramoutsaki IA, Askitopoulou H, Konsolaki E (2002). Pain relief and sedation in Roman Byzantine Texts:
*Mandragoras officinarum*, *Hyoscyamos niger* and *Atropa belladonna*. Int Congr Ser.

[32] Stannard J. The plant called moly. Osiris 1962;14:254–307. https://www.jstor.org/stable/301871

[33] Negbi M (1989). Theophrastus on geophytes. Bot J Linn Soc.

[34] Clusius C. Rariorum aliquot stirpium per hispanias observatarum historia. Plantin, Antwerp; 1576.

[35] Aedo C, Aedo C, Herrero A, Quintanar A (2013). *Leucojum* L. Flora iberica, 20.

[36] Valdés B, Nieto Feliner G (1993). *Matthiola* L. Flora iberica, 4.

[37] Nieto Feliner G, Nieto Feliner G (1993). *Erysimum* L. Flora iberica, 4.

[38] Font Quer P (2005). *Cheiranthus cheiri*. Plantas medicinales - El Dioscórides renovado.

[39] Fortes Fortes J (1980). Los fitónimos griegos: estudios de lingüística y paleoetnobotánica (Doctoral Dissertation, 2 vols.).

[40] Beekes R (2010). Etymological dictionary of greek.

[41] Murray AT. Homer. The Odyssey with an English Translation by A.T. Murray, PH.D. in two volumes. Harvard University Press, Cambridge, MA.; William Heinemann, Ltd., London; 1919.

[42] Butler S. Homer. The Odyssey. Rendered into English prose for the use of those who cannot read the original. A. C. Fifield, London; 1900.

[43] Lattimore R (1967). The Odyssey of Homer.

[44] Wilson E. Homer. The Odyssey. W. W. Norton, New York; 2018.

[45] Dallman P. Plant life in the world’s Mediterranean climates. California Native Plant Society, University of California Press, Berkeley; 1998.

[46] Davis AP, Bishop M, Davies AP, Grimshaw J (2001). The genus *Galanthus* –snowdrops in the wild. Snowdrops: a monograph of cultivated *Galanthus*.

[47] Laguna E, Deltoro VI, Pèrez-Botella J, Pèrez-Rovira P, Serra L, Olivares A, Fabregat C (2004). The role of small reserves in plant conservation in a region of high diversity in Eastern spain. Biol Conserv.

[48] Forster E (1936). Trees and plants in homer. Class Rev.

[49] Petrovska BB (2012). Historical review of medicinal plants’ usage. Pharmacogn Rev.

[50] Morton AG (1981). History of botanical science.

[51] Dawkins R (1976). The selfish Gene.

[52] Mukherjee PK, Kumar V, Mal M, Houghton PJ (2007). Acetylcholinesterase inhibitors from plants. Phytomedicine.

[53] dos Santos TC, Gomes TM, Pinto BAS, Camara AL, Paes AMDA (2018). Naturally occurring acetylcholinesterase inhibitors and their potential use for Alzheimer’s disease therapy. Front Pharmacol.

[54] Berkov S, Osorio E, Viladomat F, Bastida J, Knölker H-J (2020). Chemodiversity, chemotaxonomy and chemoecology of Amaryllidaceae alkaloids. The alkaloids: chemistry and biology.

[55] Meerow AW, Snuman DA (2006). The never-ending story: multigene approaches to the phylogeny of Amaryllidaceae. Aliso.

[56] Jenks AA, Kim S-C (2013). Medicinal plant complexes of * Salvia * subgenus * Calosphace*: an ethnobotanical study of new world sages. J Ethnopharmacol.

[57] Obón C, Rivera D, Verde A, Fajardo J, Valdés A, Alcaraz F, Carvalho AM (2012). *Árnica*: a multivariate analysis of the botany and ethnopharmacology of a medicinal plant complex in the Iberian Peninsula and the Balearic Islands. J Ethnopharmacol.

[58] Pardo-de-Santayana M, Morales R, Pardo-de-Santayana M, Pieroni A, Puri RK (2010). Chamomiles in Spain. The dynamics of plant nomenclature. Ethnobotany in the new Europe: people, health and wild plant resources.

[59] Rivera D, Obón C, Inocencio C, Heinrich M, Verde A, Fajardo J, Palazón JA (2007). Gathered food plants in the mountains of Castilla-La Mancha (Spain): ethnobotany and multivariate analysis. Econ Bot.

[60] Baumann H. Von Lilienblüten aus minoischer Sicht. Willdenowia 2006;36:389–395. 10.3372/wi.36.36135

[61] Kandeler R, Ullrich WR (2009). Symbolism of plants: examples from European-Mediterranean culture presented with biology and history of art. J Exp Bot.

[62] De Castro O, Brullo S, Colombo P, Jury S, De Luca P, Di Maio A (2012). Phylogenetic and biogeographical inferences for *Pancratium* (Amaryllidaceae), with an emphasis on the Mediterranean species based on plastid sequence data. Bot J Linn Soc.

[63] Peev DR (1996). Studies on the biology or threatened plants or the Bulgarian Flora. Bocconea.

[64] McDonald A (1807). A complete dictionary of practical gardening.

[65] Ridway GRT, Tsetshladze GR (2006). Early Greek imports in Sardinia. Greek colonisation: An account of Greek colonies and other settlements overseas.

[66] Valsecchi F (1982). Le Piante endemiche della Sardegna. Boll Soc Sarda Sci Nat.

[67] Iannello C, Pigni NB, Antognoni F, Poli F, Maxia A, de Andrade JP, Bastida J (2014). A potent acetylcholinesterase inhibitor from *Pancratium Illyricum* L. Fitoterapia.

[68] Scodel R. Listening to Homer. Tradition, narrative, and audience. University of Michigan Press, Ann Arbor; 2002.

[69] Baraka A, Harik S (1977). Reversal of central anticholinergic syndrome by galanthamine. JAMA.

[70] Hort A. Theophrastus. Enquiry into Plants, 2 vols., (vol. I, 1916; vol. 2, 1926). Harvard University Press, Cambridge, Massachusetts; Heinemann, London; 1916–1926.

[71] Mooney GW (1912). Argonautica.

[72] Wellmann M. Pedanii Dioscuridis Anazarbei de materia medica libri quinque, 3 vols. (1:1907; 2:1906; 3:1914, Repr. 1958). Weidmann, Berlin; 1906–1914.

[73] Smith SA, Brown JW (2018). Constructing a broadly inclusive seed plant phylogeny. Am J Bot.

[74] WFO. World Flora Online. Published on the Internet; 2022; http://www.worldfloraonline.org. Accessed on: 07 Jul 2022

[75] Boardman J (1998). Early Greek vase painting, 11th–6th centuries BC. A handbook.

[76] Fatur K (2020). “Hexing herbs” in ethnobotanical perspective: a historical review of the uses of anticholinergic Solanaceae plants in Europe. Econ Bot.

