# Correction: The quest for Homer’s *moly*: exploring the potential of an early ethnobotanical complex

**DOI:** 10.1186/s13002-024-00658-z

**Published:** 2024-02-08

**Authors:** Rafael Molina-Venegas, Rodrigo Verano

**Affiliations:** 1 Department of Ecology, Faculty of Science, Universidad Autónoma de Madrid, 28049 Madrid, Spain; 2 Biodiversity and Global Change Research Center (CIBC-UAM), Universidad Autónoma de Madrid, 28049 Madrid, Spain; 3 Department of Classical Philology, Universidad Complutense de Madrid, 28040 Madrid, Spain

**Correction: Journal of Ethnobiology and Ethnomedicine (2024) 20:11** 10.1186/s13002-024-00650-7

Following publication of the original article it was reported that the references cited in footnotes 1 and 2 were incorrect.

Footnote 1 erroneously cited references [71], [72], and [73]. These have been corrected to references [70], [71], and [72] respectively in the following sentence: “The text of the Odyssey is from the edition of [13], other texts of ancient cited authors (sc. Theophrastus [70], Apollonius [71], Dioscorides [72]) are likewise listed in the reference list under their editors’ names.”

Footnote 2 erroneously cited reference [71] instead of reference [70] following “…in Arthur Hort’s translation of Historia Plantarum, Enquiry into Plants”.

The references given in the reference list were correct and remain unchanged.

The original article has been updated.

