# Supplementary material for: The quest for Homer’s moly: exploring the potential of an early ethnobotanical complex
Source: J Ethnobiol Ethnomed. 2024 Jan 20;20:11. doi: 10.1186/s13002-024-00650-7 (PMC10799392; doi:10.1186/s13002-024-00650-7)
Supplement: Supplementary file 1 — Additional file 1. Table S1. Native species of Mediterranean Amaryllidoideae (including the territories around the shore of the Black Sea) with white flowers, bulbs with dark-colored outer scales and reported AChE-inhibiting activity. [file 13002_2024_650_MOESM1_ESM.docx]

SUPPORTING INFORMATION

**The quest for Homer’s *moly*: exploring the potential of an early ethnobotanical complex**

Rafael Molina-Venegas & Rodrigo Verano

**Table S1.** Native species of Mediterranean Amaryllidoideae (including the territories around the shore of the Black Sea) with white flowers, bulbs with dark-colored outer scales and reported AChE-inhibiting activity.

| **Tribe** | **Genus** | **Species** | **Author** | **References** |
| --- | --- | --- | --- | --- |
| Galantheae | *Acis* | *Acis valentina* | (Pau) Lledó, A.P.Davis & M.B.Crespo | Larsen et al. 2010 |
|  | *Galanthus* | *Galanthus nivalis* | L. | Rizzi et al. 1999  Rhee et al. 2003 |
|  |  | *Galanthus elwesii* | Hook.f. | Larsen et al. 2010  Bozkurt et al. 2017 |
|  |  | *Galanthus krasnovii* | Khokhr. | Bozkurt et al. 2021 |
|  |  | *Galanthus gracilis* | Čelak. | Bozkurt et al. 2021 |
|  |  | *Galanthus woronowii* | Losinsk. | Sarikaya et al. 2013a |
|  |  | *Galanthus alpinus* | Sosn. | Emir et al. 2019 |
|  |  | *Galanthus fosteri* | Baker | Emir et al. 2020 |
|  |  | *Galanthus rizehensis* | Stern | Sarikaya et al. 2013b |
|  |  | *Galanthus reginae-olgae* | Orph. | Conforti et al. 2010 |
|  |  | *Galanthus cilicicus* | Baker | Kaya et al. 2014 |
|  |  | *Galanthus peshmenii* | A.P.Davis & C.D.Brickell | Bozkurt et al. 2021 |
|  |  | *Galanthus x valentinei* | Beck | Sarikaya et al. 2013c |
|  | *Leucojum* | *Leucojum aestivum* | L. | Larsen et al. 2010 |
|  |  | *Leucojum vernum* | L. | Larsen et al. 2010 |
|  | *Lapiedra* | *Lapiedra martinezii* | Lag. | Larsen et al. 2010 |
| Narcisseae | *Narcissus ** | *Narcissus poeticus* | L. | López et al. 2002  Rønsted et al. 2008 |
|  |  | *Narcissus papyraceus subsp. panizzianus* | (Parl.) Arcang. | Rønsted et al. 2008 |
|  |  | *Narcissus cantabricus* | DC. | Rønsted et al. 2008 |
|  | *Sternbergia* | *Sternbergia candida* | B.Mathew & T.Baytop | Orhan at al. 2011 |
| Pancratieae | *Pancratium* | *Pancratium maritimum* | L. | Abou-Donia et al. 2013  Soltan et al. 2015 |
|  |  | *Pancratium illyricum* | L. | Iannello et al. 2014 |

*** this genus also includes several species with yellow flowers

**References**

Abou-Donia, A.H., Shawky, E., El-Din, M.M.M., El Din, A.A.S., 2013. Screening of *Pancratium maritimum* L. for acetylcholinesterase inhibitory alkaloids using thin layer chromatography in combination with bioactivity staining. J. Nat. Pharm. 4, 61–66. doi:10.4103/2229-5119.110363

Bozkurt, B., Coban, G., Kaya, G.I., Onur, M.A., Ünver-Somer, N., 2017. Alkaloid profiling, anticholinesterase activity and molecular modeling study of *Galanthus elwesii*. S. Afr. J. Bot. 113, 119–127. https://doi.org/10.1016/j.sajb.2017.08.004

Bozkurt, B., Kaya, G.I., Onur, M.A., Ünver-Somer, N., 2021. Chemo-profiling of some Turkish *Galanthus* L. (Amaryllidaceae) species and their anticholinesterase activity. S. Afr. J. Bot. 136, 65–69. https://doi.org/10.1016/j.sajb.2020.09.012

Conforti, F., Loizzo, M.R., Marrelli, M., Menichini, Federica, Statti, G.A., Uzunov, D., Menichini, F., 2010. Quantitative determination of Amaryllidaceae alkaloids from *Galanthus reginae-olgae* subsp. *vernalis* and in vitro activities relevant for neurodegenerative diseases. Pharm. Biol. 48, 2–9. https://doi.org/10.3109/13880200903029308

Emir, C., Emir, A., Bozkurt, B., Ünver-Somer, N., 2019. Phytochemical constituents from *Galanthus alpinus* Sosn. var. *alpinus* and their anticholinesterase activities. S. Afr. J. Bot. 121, 63–67. https://doi.org/10.1016/j.sajb.2018.10.021

Emir, A., Emir, C., Bozkurt, B., Ünver-Somer, N., 2020. GC/MS analysis of alkaloids in *Galanthus fosteri* Baker and determination of its anticholinesterase activity. Turk. J. Pharm. Sci. 17, 36–42. https://doi.org/10.4274/tjps.galenos.2018.26056

Kaya, G.I., Uzun, K., Sarıkaya, B., Onur, M.A., Ünver-Somer, N., 2014. Phytochemical studies on *Galanthus cilicicus*. Planta Med. 80, P1L140. https://doi.org/10.1055/s-0034-1394797

Larsen, M.M., Adsersen, A., Davis, A.P., Lledó, M.D., Jäger, A.K., Rønsted, N., 2010. Using a phylogenetic approach to selection of target plants in drug discovery of acetylcholinesterase inhibiting alkaloids in Amaryllidaceae tribe Galantheae. Biochem. Syst. Ecol. 38, 1026–1034. https://doi.org/10.1016/j.bse.2010.10.005

López, S., Bastida, J., Viladomat, F., Codina, C., 2002. Acetylcholinesterase inhibitory activity of some Amaryllidaceae alkaloids and *Narcissus* extracts. Life Sci. 71, 2521–2529. https://doi.org/10.1016/S0024-3205(02)02034-9

Orhan, I.E., Yilmaz, B.S., Altun, M.L., Saltan, G., Sener, B., 2011. Anti-acetylcholinesterase and antioxidant appraisal of the bulb extracts of five *Sternbergia* species. Rec. Nat. Prod. 5, 193–201.

Rhee, I.K., Appels, N., Luijendijk, T., Irth, H., Verpoorte, R., 2003. Determining acetylcholinesterase inhibitory activity in plant extracts using a fluorimetric flow assay. Phytochem. Anal. 14, 145–149. https://doi.org/10.1002/pca.695

Rizzi, A., Schuh, R., Brückner, A., Cvitkovich, B., Kremser, L., Jordis, U., Fröhlich, J., Küenburg, B., Czollner, L., 1999. Enantiomeric resolution of galanthamine and related drugs used in anti-Alzheimer therapy by means of capillary zone electrophoresis employing derivatized cyclodextrin selectors. J Chromatogr. B Biomed. Appl. 730, 167–175. https://doi.org/10.1016/S0378-4347(99)00186-3

Rønsted, N., Savolainen, V., Mølgaard, P., Jäger, A.K., 2008. Phylogenetic selection of *Narcissus* species for drug discovery. Biochem. Syst. Ecol. 36, 417–422. https://doi.org/10.1016/j.bse.2007.12.010

Sarikaya, B.B., Kaya, G.I., Onur, M.A., Bastida, J., Ünver-Somer, N., 2013a. Phytochemical investigation of *Galanthus woronowii*. Biochem. Syst. Ecol. 51, 276–279. https://doi.org/10.1016/j.bse.2013.09.015

Sarikaya, B.B., Ünver-Somer, N., Kaya, G.I., Onur, M.A., Bastida, J., Berkov, S., 2013b. GC-MS investigation and Acetylcholinesterase inhibitory activity of *Galanthus rizehensis*. Z. Naturforsch. C J. Biosci. 68, 118–124.

Sarikaya B.B., Berkov S., Bastida J., Kaya G.I., Onur M.A., Ünver-Somer, N., 2013c. GC-MS investigation of Amaryllidaceae alkaloids in *Galanthus xvalentinei* nothosubsp. *subplicatus*. Nat. Prod. Commun. 8, 327–328. https://journals.sagepub.com/doi/abs/10.1177/1934578X1300800312

Soltan, M.M., Hamed, A.R., Hetta, M.H., Hussein, A.A., 2015. Egyptian *Pancratium maritimum* L. flowers as a source of anti-Alzheimer’s agents. Bull. Fac. Pharm. 53, 19–22. https://doi.org/10.1016/j.bfopcu.2015.02.002
